# On Feticide
*This article is the first of a series which will form a system of Medical Jurisprudence, the want of which in our language, comprising an account of the English laws, is daily regretted by medical practitioners. The treatises we already possess, are too concise, to be of much real utility on the most important occasions, and are too imperfect, to merit the appellation of systems. In the construction of that here proposed, the writer will lay under contribution the best of those systems which have appeared in other nations, as well as all the most important dissertations which have been produced on several of its distinct branches. Abstracts of the laws of England will be given on every subject; and, when it seems advisable, those of other modern nations will be adduced; as well as such of those of different ages as may be interesting and useful. All the most important existing information will be collected, and so arranged as to furnish a clear exposition of the different relations of each subject, both in a medical and legal view; so that the physician may be well instructed respecting his own enquiries, and prepared to give his evidence in a court of justice with that precision which can only be effected by having had a previous knowledge of the laws regarding the subject on which he is to be examined. By avoiding all diffuse moral reflections, and such as must immediately occur to every person of common intelligence, it is expected that this object may be effected without intruding to a great extent on the pages of this Journal, or permitting a very considerable period to elapse before it is realized. The Criminal Code is here commenced; with those subjects relating more especially to Social Compacts merely, will follow: Political Hygiene will terminate the work.


**Published:** 1820-01

**Authors:** 


THE LONDON
Medical and Physical Journal.

1 OF VOL. XLIII.] JANUARY, 1820. [NO. 251.

"For many fortunate discoveries in medicine, and for the detection of numerous "errors, the world is indebted to the rapid circulation of Monthly Journals; "and there never existed any work to which the Faculty in EUROPE and "AMERICA were under deeper obligations than to the Medical and Physical "Journal of London, now forming a long, but an invaluable, series." RUSH.

### Original Communications.

4625

### MEDICAL JURISPRUDENCE.

ARTICLE I.—*On Feticide.**

THERE are two series of measures by which the destruction of the life of the fœtus may be effected, which it is necessary to distinguish in a physiological view of this subject; although no difference, with regard to their criminality, is recognized by the English laws: 1°, those which immediately cause its death, or which prevent the natural process of its development; 2°, those which act primarily on the mother, and indirectly destroy the life of the fœtus, either by suppressing the vital continuity between the placenta and the uterus, or by exciting the latter to contraction at a premature period,

* This article is the first of a series which will form a system of Medical Jurisprudence, the want of which in our language, comprising an account of the English laws, is daily regretted by medical practitioners. The treatises we already possess, are too concise, to be of much real utility on the most important occasions, and are too imperfect, to merit the appellation of systems. In the construction of that here proposed, the writer will lay under contribution the best of those systems which have appeared in other nations, as well as all the most important dissertations which have been produced on several of its distinct branches. Abstracts of the laws of England will be given on every subject; and, when it seems advisable, those of other modern nations will be adduced; as well as such of those of different ages as may be interesting and useful. All the most important existing information will be collected, and so arranged as to furnish a clear exposition of the different relations of each subject, both in a medical and legal view; so that the physician may be well instructed respecting his own enquiries, and prepared to give his evidence in a court of justice with that precision which can only be effected by having had a previous knowledge of the laws regarding the subject on which he is to be examined. By avoiding all diffuse moral reflections, and such as must immediately occur to every person of common intelligence, it is expected that this object may be effected without intruding to a great extent on the pages of this Journal, or permitting a very considerable period to elapse before it is realized. The Criminal Code is here commenced; with those subjects relating more especially to Social Compacts merely, will follow; Political Hygiene will terminate the work.

so as to produce what is termed *abortion.* There are measures which cannot be precisely arranged under either of these heads, as their deleterious influence may be simultaneously exerted on the mother and fœtus; such as excessive blood-letting, and abstinence from food, which tend to deprive the fœtus of the necessary supply of vital fluids. Of the first class are, instruments passed into the uterus, by the vagina, directly doing fatal violence to the fœtus; contusions on the abdomen of the mother, or firm constriction of this part; and the evacuation of the fluid contained in its enveloping membranes. Of the second class are, various drugs acting as violent emetics or cathartics, or exciting especially the action of the uterus,—as emmenagogues; irritating substances introduced into the vagina, exciting the action of the uterus immediately, or doing violence to the orifice of that organ; and, lastly, excessive depletion of the system, either by blood-letting, constant purging, or want of nutriment.*

There is a notion prevalent amongst the vulgar, that abortion may be easily and certainly produced by various drugs administered to the mother; but all potions of this kind spoken of by authors are extremely uncertain in their operation and efficacy;† and others are attended with so much danger to the mother,‡ as was remarked by Ovid§ when they were in common use by the Roman women, that they are not often resorted to. This crime is but rarely committed without advisers, who, aware of the consequences, have feared to become accomplices in an act, involving either the death of the mother, or the permanent destruction of her health. In most of the cases that have become the subject of medical or forensic enquiry, it has been found that the measures used were consonant to the indications appertaining to the first class,—that is, to express feticide; amongst which, the introduction of instruments into the uterus has been the one most frequently employed;‖ and the well-founded dread which the ordinary

---

* Tortosa, *Instituzioni di Medicina Forense,* vol. ii. p. 240.

† See Somer. *Misc. Nat. Curios.* D. i. an. vi. obs. 106. Bartholinus, *op. cit.* Dec. ii. an. i. obs. 52. Haller, *Elem. Physiol.* t. viii. p. 428. Zacutus Lusitanus, *Prax. Admir.* obs. 34, 44. Guarinonius, *Cons. Med.* c. 136. Fodere, *Med. Leg.* t. ii. p. 18. Albrecht, obs. 156, Dec. i. an. iii. Hebenstreit, *Antropologia For.* p. 377. Boemer, *Institut. Med. Leg.* Littmann, *Medicin. Forens.* cent. vi. c. 43; and Mahon, *Médecine Legale,* t. i.

‡ See Brendelius, *Obs. Med.* Dec. ii. obs. 7. Erndelius, in *Schurig. Embrilog.* p. 359; Guy-Patin, *Epist. Med.* ep. 191; Mahon, *Medicine Legale,* t. i. p. 226. See also Hebenstreit, *Antropologia For.* p. 337; Ludwig, *Med. For.* p. 89; Plouquet, *Comment. super Homicid.* p. 353; Eschembach, *Med. Leg.* p. 47; Plenck, *Med. For.* p. 66.

§ *Amorum,* l. ii. eleg. xiv.

‖ See Bianchi, *De Naturali in Hum. Corp. Vitiosa Morbosaque Gener.* p. 64. Haller, *Prel. Med. Leg.* t. i. p. 146; Sandifort, *Obs. Anat. Pathol.* l. iii. p. 2.

abettors in this crime have of the use of cutting instruments, have happily prevented this mode being often put in practice in England.

It is more proper for medical readers to turn to the sources already noticed for information on this part of the subject, than that the measures alluded to should be here described. I therefore pass on to the next point for consideration, which supposes that abortion has taken place, and that the decisions of the medical practitioner respecting it are required.

The things to be determined are, whether or not a fœtus has been expelled; and, if so, whether it has occurred before the proper period, and by whom; if the expulsion has taken place from expressly natural causes, or if it has been produced by criminal measures; and, lastly, if the fœtus was, or was not, endowed with life at the time those measures were used.

To decide the first question, it is indispensably necessary that the expelled body be examined; because, unless this be effected, or the pregnancy of the woman satisfactorily proved, although the signs of abortion be evident, the penalty of feticide cannot be attached to her;* as all the signs, as far as the mother is concerned, may be similar, whether the mass expelled from the uterus be a fœtus, or a more or less organized substance, termed a *mole*. In order that this examination may be made with sufficient accuracy, and so as to lead to a precise decision,

* "*An abortus vere factus fuerit, determinari nequit, nisi per inspectionem exactam ejus quod pro abortu habetur, si fœtus humanus reapse deprehenditur, tum res ipsa loquitur. Si, quod fieri solet, ejectum fuit amissum, corpus delicti deest, et res in suspenso manebit.*"—Plouquet, *Comment. super Homicid.* p. 362. This must be the judgment of every medical practitioner; though there exists an English law which makes the administration of drugs, or the use of any other means, with the intent of producing miscarriage in a woman "proved to be quick with child" highly penal.

Now, the highest authorities assert, that it is not possible to prove that a woman is with child, or has been with child, unless there is evidence of a fœtus having been expelled by her. It may be prudent to adduce some opinions on this subject, in addition to that of Plouquet. Zacchias, after collecting the decisions of the most celebrated physicians from Hippocrates to his own time, says, "*Ex hac ergo tantorum virorum hæsitatione conclusionem jam ratam habeas, non posse infallibiliter cognosi pregnantiam, sed solum probabiliter.*"—*Questiones Medico-legales*, t. iii. n. 15. Van-Swieten, after a similar investigation of this subject, says, "*Notandum est, magna hic prudentia opus esse medico, ne facile graviditatem vel affirmet, vel neget,* PERITISSIMI ENIM DECEPTI FUERUNT TOTIES; *nunquam magis periclitatur fama medici, quam ubi agitur de graviditate determinanda.*"—*Comment. Boerhav.* tom. vi. p. 330. Baudelocque asserts, that "*Ces symptomes qu'on appelle signes rationelles de grossesse, ne la caracterisent cependant que d'une maniere très incertaine.*"—*L'Art des Accouchements*, tom. i. p. 180. Levret, Smellie, and the best later English practitioners in midwifery, will be found to express the same sentiments; and the latest writers on Medical Jurisprudence, after collecting all the authorities on the subject, concur with them. See Tortosa, *Instituzioni di Medicina Forense*, tom. i. pp. 169-176; Mahon, *Medecine Legale*, tom. i. p. 156; Fodere, *Medecine Legale*, tom. i. ch. vi. and tom. iv. ch. v. sect. 2.

it is necessary that the first appearance of the fœtus, and its successive gradual development, be well understood: and such information may, on some occasions, be usefully connected with the history of some part of the woman's previous conduct.

From the time of the first evidence of impregnation to the fifteenth day, the product of conception appears only as a gelatinous, semi-transparent, flocculent mass, of a greyish colour, liquifying promptly, and presenting no distinct formation, even by the aid of the microscope.† At thirty days, it has the size of a large ant, according to Aristotle. In a few days more, it acquires the bulk of a barley-corn, as Burton states, or, according to others, of a common fly; Baudelocque says, only of that of the malleus of the tympanum: its length is at most from three to four lines, or somewhat less than a quarter of an inch. At forty-five days, the form, the lineaments of the principal organs, and the place from which the limbs are to arise, may be discerned. It has then been compared to a bee; its length is about ten lines. This, then, is the first period at which abortion can be proved: microscopic observation, I think, might with propriety be considered as invalid in forensic enquiries; especially as erroneous decisions in favour of the criminal are of but little importance, in comparison with the danger of fixing penalty on the innocent. At sixty days, all the parts are perfectly distinct; its length is then about two inches. During the third month, the organization becomes more perfected,—the features of the face become more distinct; the brain, the spinal marrow, and the blood-vessels, may be seen through the integuments; the hands are closed, and the fingers are proportionately very long; the genitals are evident; the penis and clitoris are relatively very large; the nymphæ are projecting, and the labiæ very thick: but so many circumstances,—as the constitution and vigour of the mother, and the greater or less degree of nutriment she may have taken, have so much influence on its growth, that nothing very precise can be determined on this subject.—I proceed with Professor Chaussier's observations. In the fourteenth or fifteenth week after conception, all the external parts may be perfectly distinguished, with the exception of the hair and nails. The fœtus nearly fills the cavity of the uterus; the great relative proportion of the fluid of the membranes having disappeared. The

† This is the statement of Professor Chaussier; and it agrees with all the observations that have been made on this subject, except those of Sir Everard Home, who has described the primordial organization of the fœtus as visible on the eighth day by the aid of Mr. Baüer's microscope.—See *Philosophical Transactions of the Royal Society of London, for the Year* 1817, part ii.; or, for an abstract of the observations alluded to, the xxxixth volume of the *London Medical and Physical Journal.*

head now descends towards the orifice of the uterus. About the end of the fifth month, the motions of the fœtus begin to become sensible to the mother, because it is then in contact with the parietes of the uterus. At this time, too, we begin to find some traces of fat under the integuments, where previously nothing but a sort of gelatine had been discerned : the ordinary length of the whole fœtus is about ten inches. The head had hitherto been very large in relation to the body ; but, in the course of the sixth month, the proportions seen in the infant become more nearly established. It is now however large, and soft ; the fontanelles are much expanded ; the skin is very fine, thin, pliant, and of a purple colour, especially in the palms of the hands, the soles of the feet, the face, the lips, the ears, and the breasts. In males, the scrotum is of a bright-red colour ; in females, the vulva is projecting, and the labiæ separated by the protuberance of the clitoris. The hair on the head is very thinly dispersed, short, and of a white or silvery colour. The eye-lids are closed ; the hair on the eye-brows, and the eye-lashes, but thinly scattered : the pupil is, ordinarily, closed by a membrane ; the nails are wanting, or scarcely apparent. In this month, too, the internal organs undergo a remarkable development : before this time the brain was but a soft mass, equally white throughout its whole extent ; it now presents a plane, uniform, surface ; but its consistence is so slight, that it dissolves on being touched by the finger ; the pia-mater seems only to lie over its surface, being separated with the greatest facility. The lungs are very small ; the heart voluminous, but its ventricles are not very distinct from the auricles ; the liver, very large, is situate near to the umbilicus ; the gall-bladder contains only a small quantity of a nearly-colourless serous fluid ; the meconium is small in quantity, and is found only in the coecum and a small portion of the colon. In the male, the testicles are found a little below the loins, near the lumbar vertebræ : in the female, the ovaries are small, elongated, very distinct from the uterus, and situate near the same vertebræ. The ordinary length is from thirteen to fourteen inches. In the course of the seventh month, when the vitality of the fœtus much increases, all the parts acquire more firmness : the skin assumes a rose tint ; the sebaceous follicles interspersed about it, begin to secrete a fluid which is extended over its surface, so as to form a whitish, unctuous, covering. The eye-lids are no longer agglutinated ; the pupillary membrane disappears ; or, properly speaking, it divides in the middle, so as to form the pupil. The hair on the head is longer, takes a deeper hue ; the nails acquire more firmness. The length is about sixteen inches. In the eighth month, the skin has more density, and a more clear tint; it is covered with very fine short hairs, and its sebaceous coating becomes more apparent; the nails are more firm; the hair of the head is longer; the breasts are often projecting, and a lactiform fluid may be pressed from them: in males, the testicles are frequently engaged in the abdominal ring. The cerebral substance has acquired more consistence; its interior is of a reddish colour, its surface is still white; the pia-mater becomes more adherent to it, and some of those grooves and undulations may now be perceived, which afterwards constitute the circumvolutions: those grooves are, however, very superficial; but they become more strongly impressed as the fœtus approaches to maturity. The spinal marrow, as well as the pons Varolii and medulla oblongata, acquire a remarkable consistence, and even firmness. The lungs are of a reddish colour; all the parts of the heart are more distinct; the liver preserves nearly its former relative size, but it is more remote from the navel; the fluid in the gall-bladder is of a yellowish colour, and has a bitter taste; the meconium is more abundant, and fills the greatest part of the large intestine. The length is about eighteen inches. At the ninth month, all the parts have acquired more consistence: the head is large, but it has a considerable degree of firmness; the bones of the cranium, although movable, touch each other at their sides; the fontanelles are less large; the hair is longer, thicker, and of a deeper colour; the sebaceous covering of the skin is more adherent, and the hair on the part is more apparent. In the male, the testicles have often passed the pubal ring, or are even in the scrotum; the nails are firmer, and prolonged to the extremity of the finger. At the termination of this month, the circumvolutions are more numerous on the surface of the brain; the parts afterwards termed the cineritious portions begin to be distinguished by their colour; the firmness of the medulla oblongata and spinal marrow is increased; the cerebellum, as well as all the basis of the cerebrum, especially the points of it corresponding to the nerves, have acquired a very remarkable consistence; whilst the mass of the lobes of the cerebrum, and all their convex surfaces, preserve much of their former softness and flexibility. The ordinary longitudinal diameter of the head, that is, from the forehead to the occiput, is from four inches to four inches and a half; and the transversal, about three inches and a half or four inches. The lungs have now become redder and more voluminous; the branch of the pulmonary artery communicating with the aorta, termed the *canalis arteriosus*, has a considerable capacity, and its parietes are more thick and dense than formerly. The foramen ovalis in the septum of the auricles of the heart, is also very large; but the valve which is to close it after birth, has acquired more firmness and more extent than at an earlier period: the liver has also more consistence, the bile is more bitter; the meconium fills the whole of the large intestine; the bladder contains urine; and, finally, every thing announces that the organs are sufficient for the performance of the functions necessary after birth.

The researches of Professor Chaussier also show, that a well-formed infant, born at the full period, ordinarily weighs about six pounds and a half; and that its length is about twenty-one inches.

The foregoing account must only be considered as a general guide in particular cases; the diversity in the dimensions and appearance of infants born at the full period, varying in a very considerable degree, as well as the progress of their development.

The researches of M. Beclard on the formation of the bones of the fœtus, furnish also some useful observations on this subject, and perhaps more precise data than those above described, as being dependant on more certain laws of the animal economy. After two months have elapsed from the period of conception, M. Beclard states, that the skeleton is about 4 inches and 3 lines in length, that of the spine 2 inches. At three months, the former is 6 inches, and the proportion of the spine as $2\frac{2}{3}$ to 6. At four months and a half, it is 9 inches, and the spine 4; at six months, 12 inches, the spine 5; at $7\frac{1}{2}$ months, 15 inches, the spine $6\frac{1}{3}$; at nine months, or the period of birth, it is ordinarily from 16 to 20 inches in length, or, at a medium, 18; and the spine is in the proportion of $7^{3}$ to 18 to the whole length of the body. These calculations were made from observations on about fifty fœtuses, at each of the above-indicated periods.

Each vertebra, consisting originally of a section of a solid cylinder, and a ring furnished with several apophyses, is in general formed by three primitive points of ossification: the one anterior, which, by its development, forms the body or solid part of the bone; and two lateral ones, which constitute the apophysarial masses, and which, uniting together and with the former, constitute the annular structure. Besides these, each vertebra is completed by several secondary points of osseous development.

At about the sixth month of intra-uterine life, two points of ossification are found in the second cervical vertebra, one situated above the other. Towards the seventh month, the superior point, which answers to the odontoid process, is larger than the inferior, which relates to the body of the bone. At about the eighth month, the transverse processes have begun to ossify in the first of the lumbar vertebræ. At the time of birth, ossification has commenced in the body of the first cervical vertebra, and also in the first bone of the coccyx. At this age, the body of the fourth lumbar vertebra, which is the most voluminous, is three lines in depth and six lines in breadth. At the same period, the lateral portions of the six superior dorsal vertebræ begin to unite together, so as to form a ring posteriorly to the bodies of those bones. The lateral arch of the second, which is the largest, forms a chord of seven or eight lines.*

In addition to the above observations, I may add two by Dr. FARR,† that, "in a fœtus of five months, the orbits of the eyes are entirely formed into bony sockets; and, in one of seven months, the small bones subservient to the organ of hearing are so perfect, as scarcely to differ from those of a complete child."

The above considerations will be of utility in various respects; especially in determining the questions, whether the expelled body be a fœtus; the probability of the period of intra-uterine life; and in the deciding whether or not, in the more advanced stages of gestation, the expulsion should be considered as abortion, or delivery at the full term of an infant of unusual smallness, &c.

Supposing the discovery of an aborted fœtus; the next question,—by whom was it produced? is, when concealment is artfully intended, often one of extreme difficulty; and it requires the utmost circumspection in the medical enquirer.

The examination of the woman alone, especially when mechanical violence has not been used, will but very rarely furnish satisfactory information, except when abortion has occurred very near the full term of gestation; and, when the examination can be made within a day or two after it had taken place, nothing short of the detection of the placenta in the uterus or vagina can, it seems evident from the doubts expressed on every sign distinctly, and on all collectively, by the best authorities, when the gestation has been of less than five or six months' duration, be considered a sufficient ground for an absolute statement that abortion has occurred.

It is always impossible, too, to prove with certainty the existence of previous pregnancy; as dropsy, and other affections, so nearly similate it on many occasions. These remarks apply to the examination of the state of the woman alone; but, in connexion with certain circumstances attending the discovery of an aborted fœtus, less strongly-marked physiological evi-

---

* *Nouveau Journal de Medicine*, tom. iv.; or *London Medical and Physical Journal*, No. 245, p. 2, *et seq.*

† *Elements of Medical Jurisprudence.*

dence of the fact might be considered as sufficient grounds for the attachment of criminality in a court of justice.

The difficulty attending the determination of abortion in a woman, is greater in proportion to the shortness of the period from conception,* and the length of time that may have elapsed after the occurrence of the act.† The having previously borne children will add, too, to the difficulty. The most forcible signs of recent delivery, especially at an early period of gestation, may be simulated, Plouquet remarks, by profuse menstruation, or rather by uterine hæmorrhage; especially if joined with a laxity of the genitals, and any tumefaction of the mouth of the uterus from other causes. The escape of a lactiform fluid from the breasts on pressure, and all the other affections of these organs noticed by authors, are of but little moment, since they appear when conception has never taken place. The wrinkled abdomen will appear after the removal of ascites; and it is constantly present, in some degree, in women of a lax habit of body, and who have borne many children. The most strongly-marked signs of abortion noticed by authors are, the evacuation of the lochiæ, which are generally composed of clots of blood mingled with grumous fluid, whilst the menses have the appearance of a dissolution of grumous blood in water, without coagula. The discharge of the lochiæ is attended with a depression of strength; whilst that of the menses gives vigour to the functions. Tumidity and redness of the pudenda, the labiæ being at the same time lax to the touch; great amplitude of the vagina; inordinate extension of the mouth of the uterus, which has also a flattened form; a loose and wrinkled state of the abdomen; a pendant, flaccid, state of the breasts, with the flow of a milky fluid from them, if the pregnancy was much advanced; vacillation in walking; and pains, especially if accompanied with action of the abdominal muscles, similar, in their forcing-downward character, to those of labour; the inferior extremities are sometimes tumefied; the veins, formerly prominent on the skin, disappear; and the external surface of the body is pale. Lafosse‡ says, the whole of these signs existing together to a certain extent, are decisive; but that the greater part of them may be simulated by some diseases.

To the remarks on the indecisive character of the greater

---

* *Eo minus evidentia erunt quo mineris molis fetus erat.*—Plouquet, *Comment. super Homicid.* p. 363. *Quo minor fœtus est, eo obscuriora partus celebrati signa esse solent.*—Hebenstreit, *Antrop. For.* p. 384.

† *Postquam suo levatus onere uterus est, ad pristinam revertitur angustiam; ut signa partus celebrati aliquo post tempore deleantur.*—Hebenstreit, *op. cit.* p. 394.

‡ In the *Encyclopédie Methodique.*

part of those signs already adduced from Plouquet, we may add the testimony of GAVARD* respecting those to be drawn from the mouth of the uterus. Nothing, he says, is more uncertain; because similar alterations may depend on a very different cause from that of delivery,—as some organic disease; and because there have been individuals, still in the state of virginity, in whom the primitive form of the uterine orifice was the same as that of a woman who had borne children.

The next question,—whether the expulsion of the fœtus has taken place spontaneously, or has been effected by criminal means, is often more easily decided; supposing the delivery of an aborted fœtus, and the detection of the mother. But, as a great proportion of the measures indicated as those usually resorted to, to procure abortion, may have been used with other intentions, some enquiries are necessary, in order to discover the motives with which they have been employed. Here, again, the court of justice must decide on the value of the evidence; but the principal points for medical consideration are the following: if the mother had a fall, or suffered any other accidental violence; if such an injury was followed by pains in the belly, discharge from the vagina, &c.; if the woman is of such an irritable temperament, that such an accident might be suspected to produce those effects; if she has lifted any great weight, or used any straining exertions, and if only a short period elapsed between those and the abortion; if any internal cause of hæmorrhage existed; if the latter and the abortion have been preceded by an evident plethoric state; if the woman has ordinarily worn very tight corsets, &c.; if, only suspecting an obstruction of the menses, she has had recourse to the use of medicines; if the woman has suffered hæmorrhage during pregnancy to a great extent, and at different periods; if the woman has shown no irregularity of conduct; if the fœtus shows no marks of violence, excepting those of contusion, in the case of a fall on the abdomen on the part of the mother, or accidental blow on that part, although it may be shrivelled, and generally livid; if the mother presents no extraordinary morbid appearances; and if, being a married woman, she has candidly made her state known to her acquaintance or medical attendant: there exist no rational grounds for suspicion of criminality. It should also be borne in mind, as Foderé remarks, that a woman may be pregnant, and arrive at the period of four or five months' gestation, without being conscious of the nature of her situation.†

On the contrary, if the woman has been known to make en-

---

* *Traité de Splanchnologie,* p. 520.

† *Méd. Legale,* t. iv. p. 437.

quiries respecting the means of procuring abortion; if she has wilfully used extraordinary exertions, and such as, aware of her state, she must know to be improper; if, although in health, she has prepared particular measures for accommodation for other illness than that attending natural delivery; if there are about her numerous recent cicatrices from venesection, or it is proved that she has required to be bled repeatedly by others than regular medical practitioners, or by those without informing them of her state, or her having been one or more times bled before, (though, it should be understood, abortion is not so certain a consequence of excessive blood-letting as Boerhaave and Van-Swieten, after Hippocrates, assert; since Baudelocque mentions cases of women who had been bled even eighty and ninety times, for some diseases, who went to the full term, and produced living infants;*) if she has endeavoured to obtain drugs commonly supposed to provoke abortion; if she has taken any violent medicines secretly; if such medicines are found in her possession; if she presents any extraordinary appearance of a morbid state of body not arising from evident causes, and which she has not made known to proper persons,—as great extenuation of strength, or squallidness; if she has suffered uterine hæmorrhage, or other discharge, to a great extent, and has not made it known; if menstruation has ceased, and her belly acquired a size disproportionate to the general appearance of her body, which she had endeavoured to conceal rather than to make her state known, especially if a married woman; if there are marks of wilful violence about the genitals, or of contusions on the belly, without any known accidental cause for them; or the fœtus presents marks of unnatural violence; or if, with some of the foregoing circumstances, the fœtus is plump and healthy in appearance, and delivery has taken place suddenly, without previous illness of the mother, or known accident rendering its occurrence a probable consequence, and there are reasons for believing that instruments of a certain kind, referred to on a former occasion, have been employed; or, lastly, if the fœtus presents marks of unnatural violence: these presumptions strongly indicate, if they are not equivalent to a proof of, criminality. The whole of them, however, cannot be expected to exist in any particular instance, and several of them, singly considered, are not decisive evidence; but, when many of those most clearly indicative of guilt are combined, the physician may give his evidence in favour of criminality with sufficient confidence.†

* *Maladies des Femmes Grosses*, l. i. c. 11.

† Bockius, *De Judicio circa Abortum concitatum Ferendo*; Lecieux, *Considerations Medico-legales sur l'Infanticide*; Schirmer, *De Lesionibus externis Abortivis, Veneris ac Philtris*; Plouquet, *Comment. super Homicid.*; Alberti, *De Abortus*

Let us, however, bear in mind the remarks of FODERE on this subject: "It appears (he says) that we should always have these two truths before our eyes, when the reality of the crime of abortion is to be verified: 1°, that if the development of the uterus has taken place in the ordinary order, there are but few means for overthrowing it, and hastening the instant of its general contraction; 2°, that if, on the contrary, this order has not existed, or the mother has been afflicted with some of the complaints capable of effecting abortion, this accident may be very natural, although preceded and accompanied by circumstances which make it appear criminal."* In confirmation of these sentiments, he cites several cases, from good authorities, of the most violent attempts having been employed without success.

PLOUQUET makes an observation,† that may with propriety be adduced in this place, although the efficacy of the measure designated may be well doubted: "*Pronum est colligere fortiores ictus electricos idem efficere, et sic fœtum in utero interimi posse, cujus rei vix ac nec vix quidem signa dabuntur.*"

As there are commonly aiders and abettors in the commission of this crime, and as the means used may produce the death of the mother, the medical practitioner may be required to deduce his evidence from examination of the dead body, as well as from the circumstances already detailed: but on this point it is not necessary to make many particular remarks, since those already adduced apply to the most important circumstances respecting it. The value of the evidence deduced from inspection of the uterus must vary much, according to peculiar circumstances; and, when death has not taken place until one or two or more days have elapsed from the time of abortion, it can but rarely furnish any decisive information. In cases of death ensuing immediately after the act of abortion, when the pregnancy has been as much as four or five months advanced, the appearances of the uterus then might furnish some important evidence, when added to other concomitant circumstances. The most decisive of those appearances are, the greater than natural thickness and capacity of the uterus; the traces of the adhesion of the placenta; the inequalities of its surface, the distension of its neck, and the dilatation of the vagina throughout its whole extent; and, if the opening be made very soon

*violenti Modis et Signis;* FARR's *Elements of Medical Jurisprudence;* TORTOSA, *Instituzioni di Medicina Forense*, vol. ii. pp. 249, 258; MAHON, *Méd. Légale*, t. i. p. 225.

* *Médecine Légale*, t. ii. p. 15. See also the authorities cited in the note (†), page 2.

† *Commentatio super Homicid.* p. 346.

after death, and the pregnancy was considerably advanced, it is probable that the venous sinuses may still remain considerably enlarged.*

The last question,—whether or not the fœtus was endowed with life at the time the criminal means were used, supposing these to have been substantiated, although insignificant in a medical point of view, is of great importance in relation to the laws of England, by which a difference is made in the penalty attached to the crime of procuring abortion, if the woman be "quick with child," and before she is "quick with child." This I shall take into consideration in another part of this essay. In the eyes of the well-informed physiologist, such a distinction is founded on error. "*Semper enim,* (says HEBENSTREIT,) *ex quo in minimo stamine ovuli materni crescere fœtus incipit, vivit, adeoque animam semper habet. Nullum tempus est quo fœtus homo dici nequeat, et anima, simul ac fœtus crescere incipit, in minimo etiam exemplo adest.*"†—PLOUQUET expresses the same opinion: "*Vita inchoat in ipso conceptionis momento in embryone nondum visibili, adeoque destructio vel prœmatura expulsio ejus sensu philosophico semper et in quocumque graviditatis termino homicidio aequiparanda erit.*"‡ Similar sentiments have been expressed by various other estimable authorities; and TERTULLIAN agrees with them, and indicates that it should be so considered in jurisprudence: he says, "*Homicidii festinatio est prohiberi nasci. Nec refert natam quis eripiat animam, an nascentem disturbet. Homo est qui futurus est.*" §

As there are no certain signs of the death of the fœtus in the uterus, it must, in the eye of the physician, be considered alive until its expulsion, unless there is then evidence of the contrary; but, when this exists, it will often be impossible for him to determine whether or not death took place previously to the use of the criminal measures; and these may at best be but presumptive evidence. However, if cutting instruments, or other mechanical violence, have been proved to have been employed, and the fœtus bears the marks of them, appearing in other respects to have been healthy and properly formed, there are, perhaps, sufficient grounds for attributing death to the criminal means.

On the contrary, a great depression of the fontanels without

---

* See WALDSCHMID, *Dissertatio de Abortus facti Signis in Matris præsertim defunctæ partibus Generationi inserventibus reperiundis,* Kilon. 1723; the different publications on the Trial of Charles Angus, in the years 1808-1809; and the discussion on that subject in the *London Medical and Physical Journal,* vol. xxi. pp. 336, 343, 399, 426, 439.

† *Antropolog. For.* p. 374.

‡ *Comment. super Homicid.* p. 367.

§ *Apologetic,* c. ix. p. 550.

external violence; the separation of the epidermis in several places; a blue and livid colour of the skin; a cadaverous odour; a shrivelled, softened, and almost pultaceous, state of the muscles and viscera; a shrunken and tender state of the umbilical cord, and an emphysematous state of the body generally, or of the abdomen, are strong evidences that the fœtus perished a considerable time before its expulsion: but it may be impossible to determine the exact time of its death, or whether or not it died previously to the use of means calculated to produce it. If those measures had not been put in practice until a few days before abortion, and the mother declared that she had not felt the motions of the fœtus for some days previously, it will be prudent for the physician to give his evidence in a manner the most favourable to the mother. When the examination of the fœtus is not made until many days have elapsed since its expulsion, and it has consequently become putrid, nothing else than the appearance of wounds with instruments can afford any decisive evidence respecting the point under consideration. The questions, whether the fœtus, if endowed with life when expelled from the uterus, might have continued to live if it had been attended to with proper care; or whether violence had been done it after its expulsion, come under the head of *Infanticide*. H.

*London; Dec. 1st,* 1819.

